# Serum IRAK3 may serve as a prognostic biomarker in acute supratentorial intracerebral hemorrhage: findings from a prospective observational cohort study

**DOI:** 10.3389/fneur.2024.1436997

**Published:** 2024-09-13

**Authors:** Yijun Ma, Jun Wang, Chao Tang, Wei Li, Xuan Lv, Suijun Zhu

**Affiliations:** ^1^Department of Neurosurgery, First People's Hospital of Linping District, Hangzhou, China; ^2^Department of Neurosurgery, Linping Campus, The Second Affiliated Hospital of Zhejiang University School of Medicine, Hangzhou, China

**Keywords:** intracerebral hemorrhage, IRAK3, prognosis, severity, outcome, biomarkers

## Abstract

**Background:**

Interleukin-1 receptor-associated kinase 3 (IRAK3) modulates neuroinflammation. This study aimed to determine the prognostic role of serum IRAK3 in acute intracerebral hemorrhage (ICH).

**Methods:**

In this prospective observational cohort study, 152 patients with supratentorial ICH, along with 63 healthy controls, were recruited. Serum IRAK3 levels were measured at the time of enrollment for controls, at admission for all patients, and on poststroke days 1, 3, 5, 7, 10, and 15 in a subset of 63 patients. Stroke severity was assessed using the National Institutes of Health Stroke Scale (NIHSS) and hematoma volume. Poststroke 6-month modified Rankin Scale (mRS) scores were registered, with scores of 3–6 representing a poor prognosis. Multivariate models were established to investigate severity correlation and prognosis association.

**Results:**

Serum IRAK3 levels were significantly elevated at the admission of patients, peaked at day 1, plateaued at day 3, gradually declined until day 15, and were substantially higher over the first 15 days poststroke than in controls. Admission serum IRAK3 levels were independently associated with NIHSS scores, hematoma volume, and 6-month mRS scores in a multivariate linear regression model. They were linearly correlated with the risk of poor prognosis in a restricted cubic spline analysis and were independently predictive of poor prognosis in a binary logistic regression model. Additionally, they demonstrated strong prognostic ability in the receiver operating characteristic curve analysis. Using subgroup analysis, no interactions were found between admission serum IRAK3 levels and some routine variables, such as age, gender, hypertension, and diabetes mellitus. Moreover, the model combining admission serum IRAK3, NIHSS scores, and hematoma volume demonstrated stability and clinical value in calibration and decision curve analyses.

**Conclusion:**

A significant increase in **s**erum IRAK3 levels during the early phase after ICH, strongly correlated with disease severity, is independently associated with a poor 6-month prognosis, establishing serum IRAK3 as a valuable prognostic biomarker for ICH.

## 1 Introduction

Spontaneous intracerebral hemorrhage (ICH), constituting 10–20% of all strokes, is a catastrophic cerebrovascular disease and represents a global public healthcare problem (1). Following a brain hemorrhagic insult, some pathophysiological processes, such as excitotoxicity, oxidative/nitrosative stress, and inflammation, are induced, thereby damaging the extracellular matrix, disrupting cellular integrity, destroying the blood–brain barrier, causing neuronal death and finally leading to neurological deficits (2). The National Institutes of Health Stroke Scale (NIHSS) scoring and hematoma volume are the two acceptable indicators of severity and prognosis assessment in ICH (3, 4). During recent decades, some biomarkers have been noted with respect to the value of severity evaluation and prognosis prediction of ICH (5–7).

Interleukin-1 receptor-associated kinase 3 (IRAK3), also called IRAKM, has been recognized for its regulatory role in inflammatory responses, potentially by modulating Toll-like receptor signaling pathways (8–10). Studies have shown that in experimental autoimmune encephalomyelitis, IRAK3 expression is increased in microglia, and mice lacking IRAK3 exhibit significantly more severe disease symptoms (11).

Similarly, both protein and mRNA levels of IRAK3 are significantly elevated in neurons, and the deficiency of IRAK3 greatly exacerbates neuronal damage in a mouse model of sub-acute Parkinson's disease (12). Consistent with these findings, IRAK3 deficiency in experimental stroke models leads to increased infarct volume, worsened brain edema, a higher incidence of hemorrhage transformation, and compromised blood–brain barrier permeability (13).

These results suggest that IRAK3 may possess potent neuroprotective properties. Recently, it was observed that serum IRAK3 levels were significantly elevated after severe traumatic brain injury, with a strong correlation to trauma severity, and were independently predictive of poor outcomes at 6 months (14). In this study, we aimed to investigate the longitudinal change in serum IRAK3 levels following ICH and assess its potential prognostic value in a cohort of ICH patients.

## 2 Materials and methods

### 2.1 Study design and ethical approval

This observational study, conducted from January 2019 to December 2022 at the First People's Hospital of Linping District (Hangzhou, China), had two primary objectives.

The first objective was to uncover changes in serum IRAK3 levels after ICH compared to healthy controls through a cross-sectional study. The study aimed to track the dynamic changes in IRAK3 levels following ICH using a longitudinal approach, and the other objective was to unveil the prognostic significance of admission serum IRAK3 as a biomarker of ICH using a prospective cohort study. In compliance with the voluntary principle, some patients agreed to blood draws only at admission, and others consented to blood collections at admission and on days 1, 3, 5, 7, 10, and 15 after the stroke. Moreover, blood samples of controls were acquired during their recruitment into the study.

This study was conducted in accordance with the Declaration of Helsinki and its later amendments. The protocol was pre-approved by the Ethics Committee of the First People's Hospital of Linping District (NO. LPH2018012), and informed consent was obtained from the patients' next of kin or the control participants themselves.

### 2.2 Participant enrollments

All consecutively selected patients were admitted to our hospital due to ICH, which was confirmed using a head computerized tomography (CT) scan.

Patients were eligible if they were aged 18 years or older, presented to our emergency center within 24 h of stroke onset, had new-onset hemorrhagic stroke, non-secondary brain hemorrhages, and a hematoma located in the supratentorial cavity, and were undergoing conservative therapy for the hematoma. Patients were excluded if they (1) had a history of or concurrent neurological diseases, such as ischemic stroke, intracranial neoplasms, craniocerebral trauma, intracranial infections, degenerative disorders, or immune illnesses; (2) had severe comorbidities such as leukemia, chronic obstructive pulmonary disease, liver cirrhosis, chronic heart failure, or chronic renal disease; or (3) had specific conditions or medications, such as pregnancy, use of immunosuppressants, missed visits, incomplete information, refusal to participate, or unqualified blood samples.

Healthy volunteers were consecutively chosen to form a control group. These patients had no history of hypertension, diabetes mellitus, chronic renal disease, or other significant health issues and had normal results in blood tests, such as white blood cell counts, blood sugar levels, platelet counts, and red blood cell counts.

### 2.3 Data collection

Upon entry at the emergency center, patients' demographic information, medical histories, and medications were inquired, and the registered data were age, sex, cigarette smoking, alcohol drinking, hypertension, diabetes mellitus, dyslipidemia, antilipidemic drugs, anticoagulative agents, and antiplatelet therapies. Basic vital sign measurements included blood pressure, pulse rate, heart rate, respiratory rate, and blood oxygen saturation. All patients were checked using a head CT scan. The observed radiological parameters were supratentorial bleeding sites (superficial/deep), three-dimensional parameters of hematoma (height/depth/length), an intraventricular extension of hematoma, and an extension of hematoma into the subarachnoid cavity. Hematoma size was estimated using the following equation: height × depth × length × 0.5 (ml) (15). Neurological function status was appraised using NIHSS scoring at admission. Modified Rankin Scale (mRS) scores, ranging from 0 to 6, were documented at 6 months following stroke. The scores of 3–6 signified a poor prognosis (16).

### 2.4 Quantification of serum IRAK3 levels

All patients and controls underwent venous blood draws.

Blood samples were acquired at admission from patients consenting to single-time blood collections and at admission as well as on poststroke days 1, 3, 5, 7, 10, and 15 from those agreeing to multiple time point collections. Blood samples of controls were harvested at their enrollment in the study. In total, 5 ml of venous blood was obtained and put in a gel-containing biochemical tube. Blood samples were spun at 2,000 × g for 10 min. Afterward, the supernatants were separated, placed into labeled Eppendorf tubes, and preserved in a −80°C freezer for further detection. Every 3 months, a batch of collected blood samples was removed, and the serum was thawed. Using the blind method, IRAK3 levels were in duplicate gauged following the manufacturer's instructions via the sandwich enzyme-linked immunosorbent assay (commercial kit: Shanghai Jianglai Biotechnology Co., Ltd., Shanghai, China; Product Number: JL30214). The detection range varied from 0.78 to 50 ng/ml, with a minimum detectable concentration of 0.36 ng/ml. The intra-assay coefficients of variation were <8 and <10%, respectively.

### 2.5 Statistical analysis

Data were analyzed with the utilization of the Statistical Package for the Social Sciences 23.0 (SPSS Inc., Chicago, IL, USA) and R 3.5.1 (https://www.r-project.org), except receiver operating characteristic (ROC) curve analysis and sample size calculation, in which MedCalc 20 (MedCalc Software, Ltd., Ostend, Belgium) was employed. For intergroup comparisons, ROC curve analysis, or other statistical analyses, a total of 152 patients were adequate for statistical investigation. Graphs were generated using GraphPad Prism 7.01 (GraphPad Software Inc., San Diego, California, USA). Categorical variables are summarized in the form of counts (percentages). As for continuous variables, the normality test was conducted using the Kolmogorov–Smirnov test, and subsequently, those data were reported as means (standard deviations, SDs) or medians (lower-upper quartiles) in light of distribution patterns.

Statistical methods used for intergroup comparison included the chi-squared test, the Fisher's exact test, the independent-sample Student's *t*-test, and the Mann–Whitney *U*-test. Applying the Kruskal–Wallis test, we determined whether admission serum IRAK3 levels significantly differed among multiple subgroups, which were divided based on mRS scores.

The Spearman's rank correlation coefficient was employed to discern variables that were tightly correlated with admission serum IRAK3 levels and mRS scores 6 months after ICH. Afterward, those significantly correlative variables were incorporated into the multivariate linear regression model to ascertain dependent variables. Under the restricted cubic spline, we demonstrated whether there was a linear correlation between admission serum IRAK3 levels and the risk of poor prognosis. Under the ROC curve, discriminative efficiency was appraised, with the Youden method used to identify the cutoff value of admission serum IRAK3 levels. The binary logistic regression model was constructed to confirm the relationship between admission serum IRAK3 levels and 6-month poor prognosis following ICH. Moreover, the combined logistic regression model was configured to verify the prognostic additive effects of admission serum IRAK3 levels on NIHSS scores and hematoma. A nomogram graphically represented the combination model for poor prognosis. In this nomogram, different values of the above three variables corresponded to the different scoring points, and all scoring points were summed to form the total scores, which pointed to a risk of poor prognosis. The model was assessed for its stability using calibration curve analysis and for its clinical fitness using decision curve analysis. Subgroup analysis of poor prognosis was conducted to assess whether admission serum IRAK3 levels interacted with some conventional variables, such as age, gender, hypertension, diabetes mellitus, and dyslipidemia. A two-sided *P*-value of <0.05 indicates statistically significant differences.

## 3 Results

### 3.1 Participant selection and characteristics

**Figure 1:**
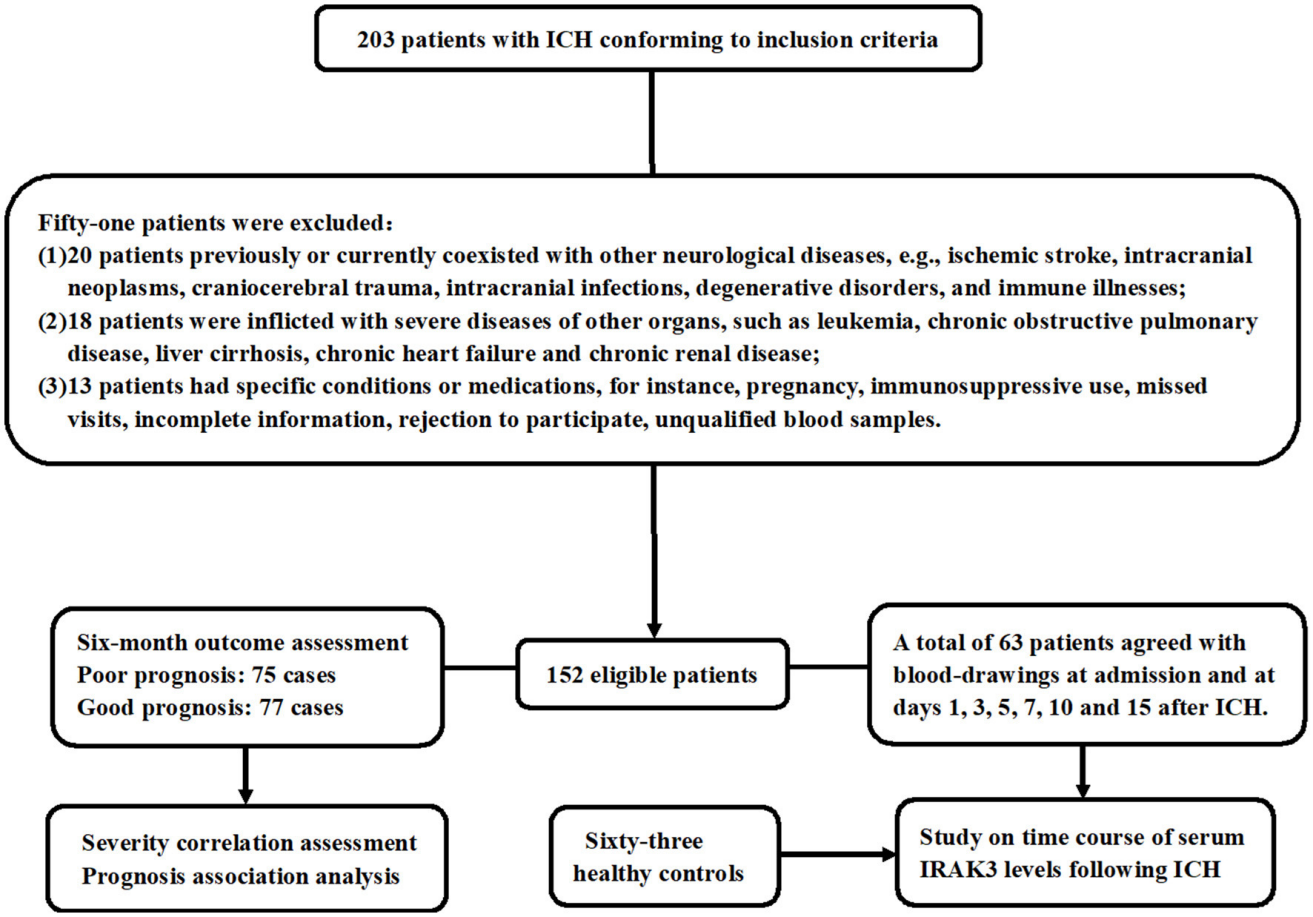
Study plan and flowing chart for clinical study of acute ICH. A total of 203 patients with ICH fulfilled the inclusion criteria and were consecutively recruited. Then, 51 patients were removed from this study because of the exclusion criteria that had previously been presented. At last, 152 patients were retained for the final clinical investigation. Seventy-five patients had a poor prognosis at 6 months following ICH, and 63 patients agreed with blood collection at multiple time points. Admission blood samples were collected for severity and prognosis-related studies, and blood samples at multiple time points were obtained to investigate the temporal change of serum interleukin-1 receptor-associated kinase 3 levels. ICH denotes ICH; IRAK3, interleukin-1 receptor-associated kinase 3.

During the study period, a total of 203 ICH patients were consecutively enrolled in compliance with the previously presented inclusion requirements. As shown in Figure 1, 51 patients were excluded based on the prespecified criteria. Ultimately, 152 patients were included in the clinical assessment. Of the 152 patients, only 63 of them consented to multiple blood draws after ICH. Meanwhile, 63 controls were selected. Those 63 patients were aged from 39 to 82 years (mean, 60.3 years; SD, 10.6 years), including 33 men and 30 women, as well as containing 19 cigarette smokers and 20 alcohol drinkers. Those 63 controls were aged from 41 to 86 years (mean, 62.7 years; SD, 12.5 years), comprised 35 men and 28 women, and consisted of 21 cigarette smokers and 22 alcohol drinkers. There were no significant differences between the 63 patients and 63 controls in mean age, gender distribution, smoking status, or alcohol consumption (all *P* > 0.05).

As for all 152 patients, age ranged from 38 to 85 years, with a mean value of 60.9 years (SD, 11.6 years). A total of 71 patients were aged ≥65 years, and 81 patients were aged <65 years. A total of 86 men and 66 women were included. In total, 96 hypertensive patients, 32 diabetic patients, and 45 dyslipidemic patients were found. A total of 53 patients were cigarette smokers, and 56 patients were alcohol consumers. Altogether, 35, 12, and 26 patients were administered orally with antilipidemic agents, anticoagulants, and antiplatelet drugs.

Patients were hospitalized between 0.5 and 24 h after ICH (median 8.3 h; lower-upper quartiles, 5–14 h) after ICH, and blood samples were collected between 1 and 25 h post-stroke (median, 9 h; lower-upper quartiles, 6.3–2.3 h).

Systolic and diastolic arterial pressures ranged from 112 to 207 mmHg and from 67 to 113 mmHg, respectively, with mean values of 140.3 (SD, 22.0 mmHg) and 83.4 mmHg (SD, 8.9 mmHg), respectively. Superficial hematomas were found in 44 patients, and deep bleedings were revealed among 108 patients. Therefore, the ratio of patients with superficial to deep hemorrhage was 1: 2.46. There were 31 patients with intraventricular extension of hematoma. Hematoma was extended into the subarachnoidal cavity among 10 patients. NIHSS scores varied from 0 to 17 (median, 9; lower-upper quartiles, 5–12), and hematoma size changed from 2 to 37 ml (median, 12 ml; lower-upper quartiles, 7–22 ml). In total, 42 patients were inflicted with early neurological deterioration, 43 patients experienced pneumonia, and nine patients presented with seizures.

### 3.2 Temporal change of serum IRAK3 levels and its relation to ICH severity

**Figure 2:**
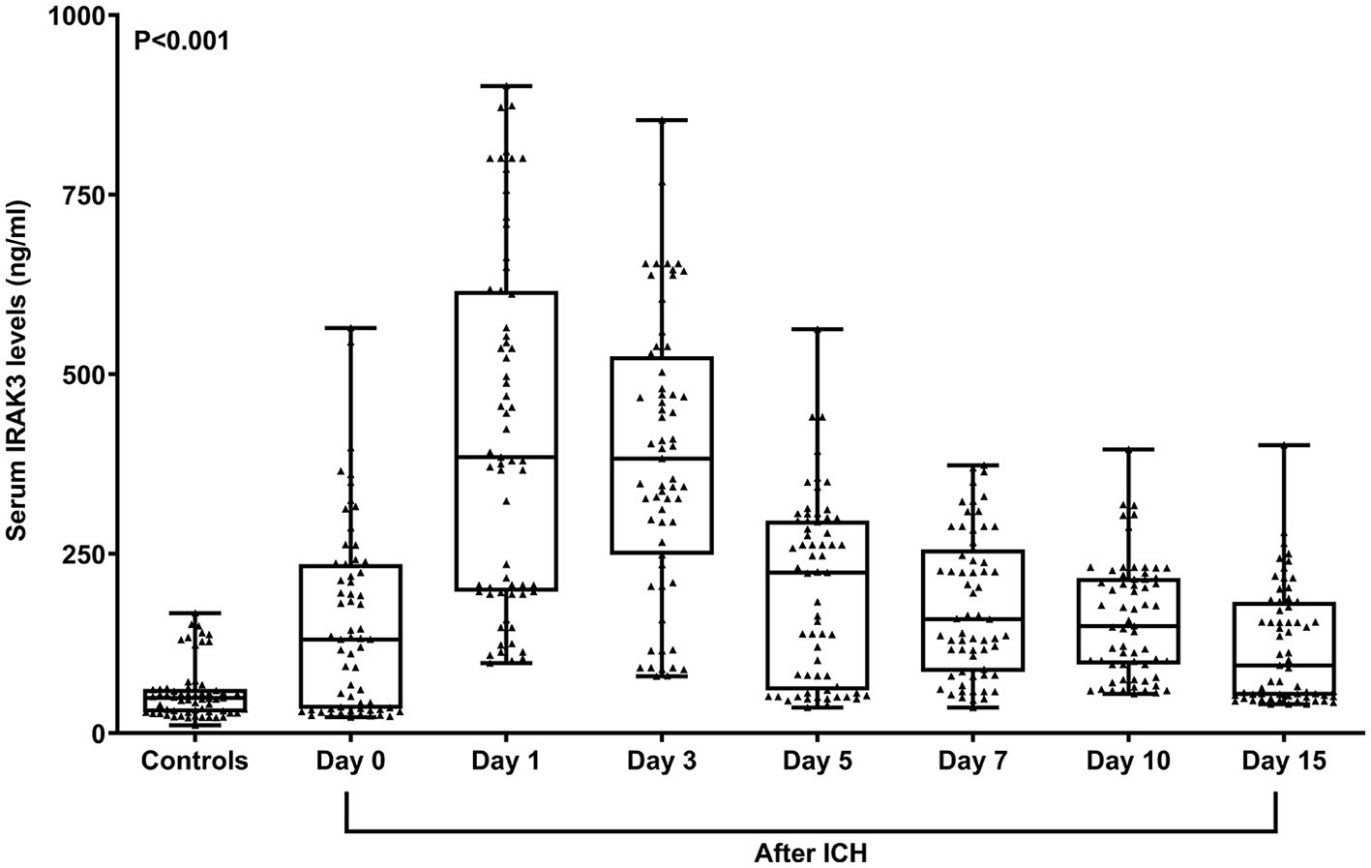
Longitudinal change of serum interleukin-1 receptor-associated kinase 3 levels following acute ICH. Serum interleukin-1 receptor-associated kinase 3 levels were immediately elevated at admission in patients with ICH, with substantially highest levels at days 1 and 3, and remained at significantly higher levels until day 15 compared to controls (*P* < 0.001). ICH denotes ICH; IRAK3, interleukin-1 receptor-associated kinase 3.

**Figure 3:**
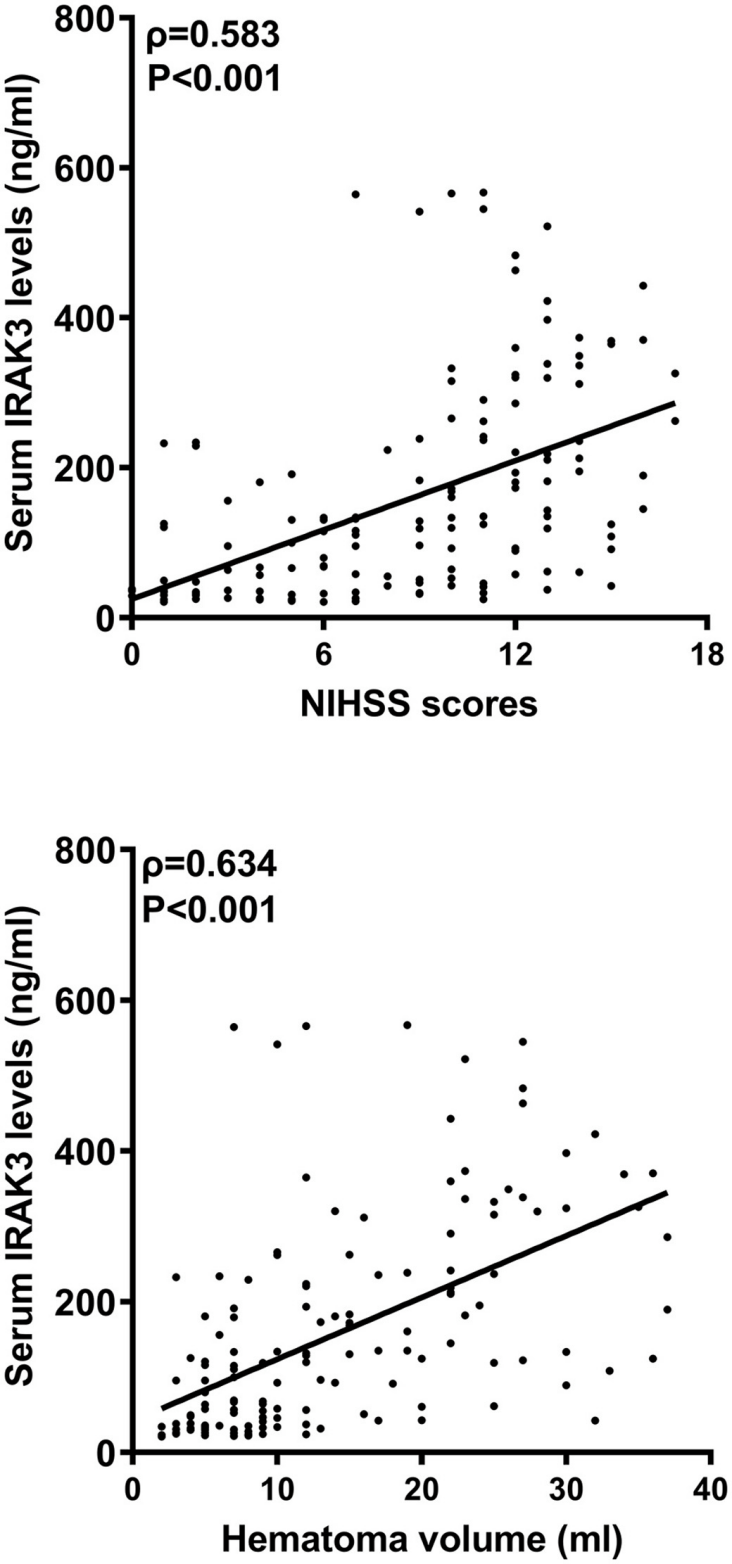
Admission serum interleukin-1 receptor-associated kinase 3 levels and stroke severity after acute ICH. Admission serum interleukin-1 receptor-associated kinase 3 levels significantly correlated with National Institutes of Health Stroke Scale scores and hematoma volume following acute ICH (*P* < 0.001). IRAK3 denotes interleukin-1 receptor-associated kinase 3; NIHSS, National Institutes of Health Stroke Scale.

In Figure 2, serum IRAK3 levels of those designated 63 patients were immediately elevated at admission, peaked on day 1, plateaued on day 3, and then gradually reduced on day 5 until day 15, as well as being substantially raised during 15 days when compared to those 63 controls (all *P* < 0.05). Alternatively, admission serum IRAK3 of all patients was intimately and positively correlated with admission NIHSS scores and baseline hematoma volume (both *P* < 0.001; Figure 3). Moreover, admission serum IRAK3 levels were tightly related to intraventricular extension of hematoma and blood glucose levels (both *P* < 0.01; Table 1). Thereafter, NIHSS scores, hematoma volume, intraventricular extension of hematoma, and blood glucose levels were incorporated into the multivariate linear regression model, and admission serum IRAK3 levels had independent correlation with NIHSS scores (beta, 6.681; 95% confidence interval, 2.288–11.075; VIF, 2.741; *P* = 0.003) and hematoma volume (beta, 5.294; 95% confidence interval, 1.974–8.615; VIF, 2.786; *P* = 0.002).

**Table 1:** Correlation analysis of admission serum interleukin-1 receptor-associated kinase 3 levels after acute ICH.

	**Spearman test**		**Univariate linear regression analysis**	
	ρ	***P*** **-value**	β **(95% CI)**	***P*** **-value**
Age (years)	0.054	0.505	0.960 (−0.977–2.897)	0.114
Gender (male/female)	0.007	0.935	−4.171 (−49.281–40.939)	0.855
Hypertension	0.005	0.953	0.682 (−45.675–47.038)	0.977
Diabetes mellitus	0.157	0.054	30.200 (−24.433–84.834)	0.276
Dyslipidemia	0.056	0.491	14.626 (−34.400–63.552)	0.556
Cigarette smoking	0.087	0.286	28.308 (−18.393–75.008)	0.233
Alcohol drinking	0.093	0.254	35.336 (−10.669–81.341)	0.131
Antilipidemic use	−0.039	0.636	−13.011 (−66.084–40.063)	0.629
Anticoagulant use	0.089	0.276	29.187 (−53.605–111.979)	0.487
Antiplatelet use	0.076	0.352	17.448 (−41.869–76.766)	0.562
Admission time (h)	−0.116	0.155	–.2.962 (−6.444–0.519)	0.095
Blood-collection time (h)	−0.101	0.215	−2.649 (−6.101–0.802)	0.131
Systolic arterial pressure (mmHg)	−0.090	0.270	−0.059 (−1.077–0.960)	0.910
Diastolic arterial pressure (mmHg)	−0.085	0.298	−0.449 (−2.960–2.063)	0.725
Hemorrhagic locations (superficial/deep)	−0.033	0.689	−1.497 (−50.803–47.809)	0.952
Intraventricular extension of hematoma	0.226	0.005	75.221 (21.067–129.375)	0.007
Subarachnoidal extension of hematoma	0.099	0.227	64.522 (−25.073–154.118)	0.157
NIHSS scores	0.583	<0.001	15.379 (11.098–19.661)	<0.001
Hematoma volume (ml)	0.634	<0.001	8.186 (6.137–10.235)	<0.001
Blood leucocyte count (× 10^9^/l)	0.147	0.071	9.971 (−0.323–20.264)	0.058
Blood glucose levels (mmol/l)	0.269	0.001	11.045 (6.199–15.892)	<0.001

NIHSS, National Institutes of Health Stroke Scale; 95% CI, 95% confidence interval; ρ, rho; β, beta.

### 3.3 Serum IRAK3 levels and poststroke 6-month mRS scores

**Figure 4:**
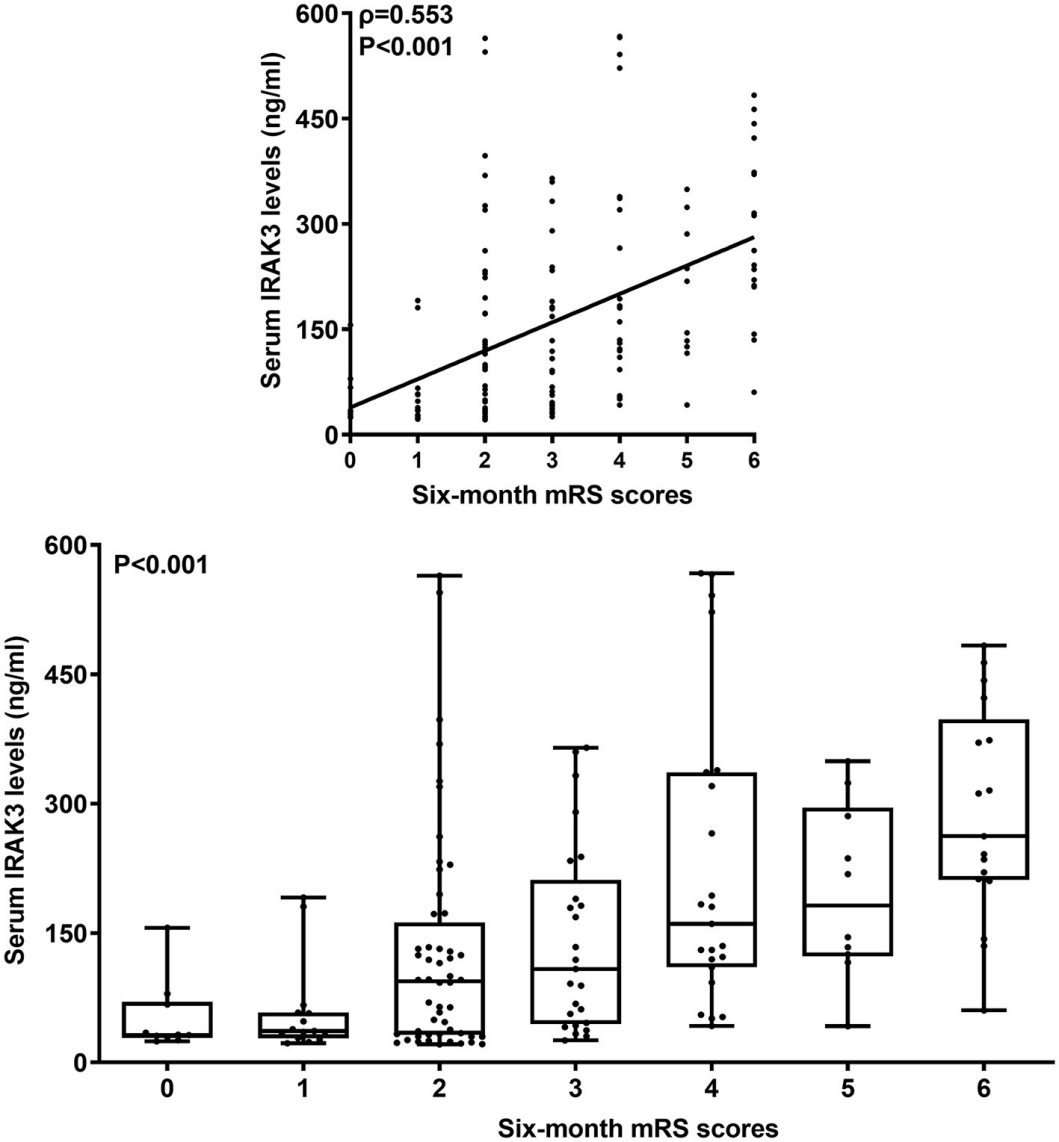
Admission serum interleukin-1 receptor-associated kinase 3 levels and modified Rankin Scale scores 6 months after acute ICH. Admission serum interleukin-1 receptor-associated kinase 3 levels were highly positively correlated with modified Rankin Scale scores (*P* < 0.001) and were substantially elevated in order of modified Rankin Scale scores at 6 months after acute ICH (*P* < 0.001). IRAK3 denotes interleukin-1 receptor-associated kinase 3; mRS, modified Rankin Scale.

Poststroke mRS scores varied from 0 to 6 (median, 2; lower-upper quartiles, 2–4). The scores from 0 to 6 were revealed in 10, 15, 52, 25, 23, 10, and 17 patients, respectively. In Figure 4, mRS scores were closely and positively related to admission serum IRAK3 levels (*P* < 0.001), and admission serum IRAK3 levels were pronouncedly raised in the order of mRS scores from 0 to 6 (*P* < 0.001). Moreover, mRS scores were prominently correlated with age, diabetes, intraventricular extension of hematoma, subarachnoidal extension of hematoma, NIHSS scores, hematoma volume, early neurological deterioration, and blood glucose levels (all *P* < 0.05; Table 2). Using the multivariate logistic regression model, in which the preceding significantly correlative variables were entered, it was demonstrated that the factors, which were independently correlated with mRS scores, were admission serum IRAK3 levels (beta, 0.002; 95% confidence interval, 0.001–0.004; VIF, 1.557; *P* = 0.011), NIHSS score (beta, 0.143; 95% confidence interval, 0.065–0.222; VIF, 2.903; *P* = 0.001), and hematoma volume (beta, 0.053; 95% confidence interval, 0.022–0.085; VIF, 1.742; *P* = 0.004).

**Table 2:** Correlation analysis of 6-month modified Rankin scale scores after acute ICH.

	**Spearman test**		**Univariate linear regression analysis**	
	ρ	***P*** **-value**	ρ **(95% CI)**	***P*** **-value**
Age (years)	0.173	0.033	0.008 (0.001–0.014)	0.032
Gender (male/female)	−0.114	0.163	−0.065 (−0.228–0.097)	0.429
Hypertension	−0.026	0.746	−0.039 (−0.206–0.128)	0.648
Diabetes mellitus	0.170	0.036	0.744 (0.100–1.387)	0.024
Dyslipidemia	0.037	0.647	0.151 (−0.024–0.326)	0.089
Cigarette smoking	0.084	0.302	0.083 (−0.086–0.251)	0.335
Alcohol drinking	−0.004	0.916	0.067 (−0.100–0.234)	0.429
Antilipidemic use	0.051	0.530	0.138 (−0.052–0.329)	0.153
Anticoagulant use	0.062	0.449	0.098 (−0.201–0.396)	0.519
Antiplatelet use	0.064	0.437	0.101 (−0.113–0.314)	0.353
Admission time (h)	−0.067	0.409	−0.010 (−0.052–0.032)	0.636
Blood-collection time (h)	−0.065	0.431	−0.008 (−0.050–0.033)	0.689
Systolic arterial pressure (mmHg)	−0.080	0.328	−0.002 (−0.014–0.010)	0.727
Diastolic arterial pressure (mmHg)	−0.043	0.597	−0.003 (−0.033–0.027)	0.865
Hemorrhagic locations (superficial/deep)	0.052	0.525	0.167 (−0.421–0.754)	0.576
Intraventricular extension of hematoma	0.224	0.006	0.959 (0.315–1.603)	0.004
Subarachnoidal extension of hematoma	0.183	0.024	1.304 (0.249–2.360)	0.016
NIHSS scores	0.594	<0.001	0.212 (0.163–0.260)	<0.001
Hematoma volume (ml)	0.578	<0.001	0.093 (0.068–0.118)	<0.001
Early neurological deterioration	0.221	0.006	0.756 (0.172–1.340)	0.012
Pneumonia	0.142	0.081	0.457 (−0.131–1.045)	0.127
Seizure	0.066	0.418	0.480 (−0.648–1.608)	0.402
Blood leucocyte count (× 10^9^/l)	0.111	0.173	0.101 (−0.023–0.224)	0.109
Blood glucose levels (mmol/l)	0.277	0.001	0.123 (0.065–0.182)	<0.001
Admission serum IRAK3 levels (ng/ml)	0.553	<0.001	0.006 (0.004–0.007)	<0.001

NIHSS, National Institutes of Health Stroke Scale; IRAK3, interleukin-1 receptor-associated kinase 3; 95% CI, 95% confidence interval; ρ, rho; β, beta.

### 3.4 Serum IRAK3 levels and poststroke 6-month poor prognosis

**Figure 5:**
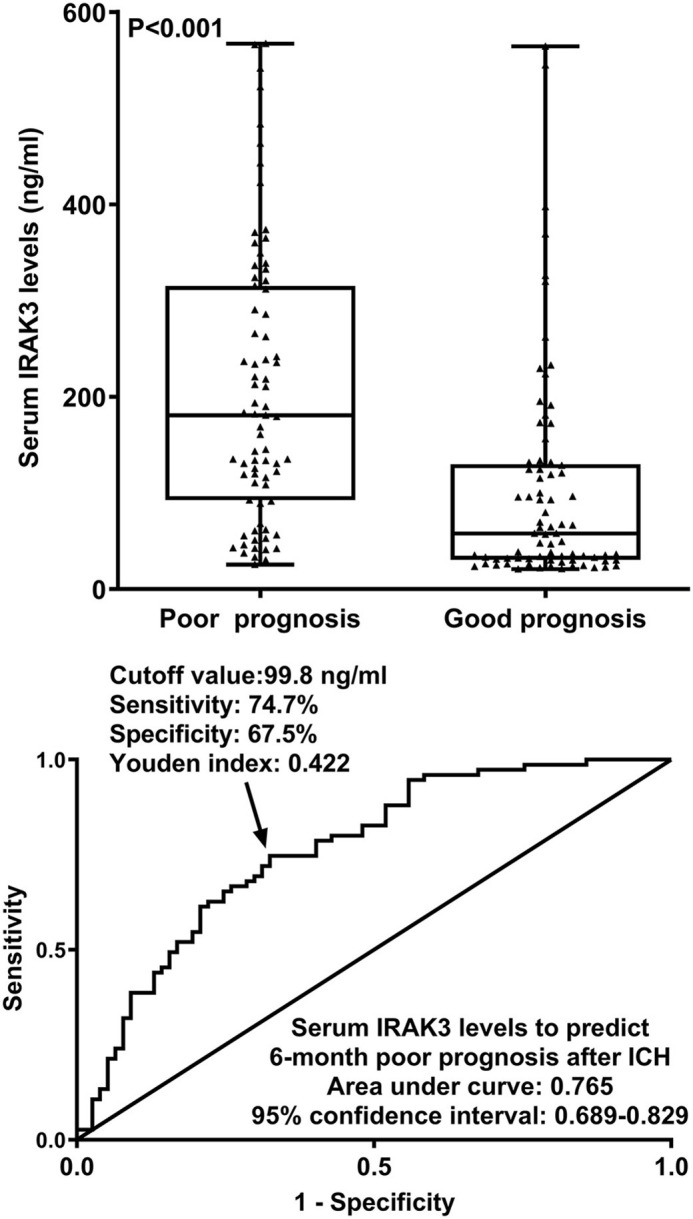
Admission serum interleukin-1 receptor-associated kinase 3 levels for predicting poor prognosis at 6 months following acute ICH. Admission serum interleukin-1 receptor-associated kinase 3 levels were dramatically higher in patients with the development of poor prognosis than in those presenting with good prognosis (*P* < 0.001) and efficiently distinguished poor prognosis at 6 months after hemorrhagic stroke. IRAK3 denotes interleukin-1 receptor-associated kinase 3; ICH, ICH.

**Figure 6:**
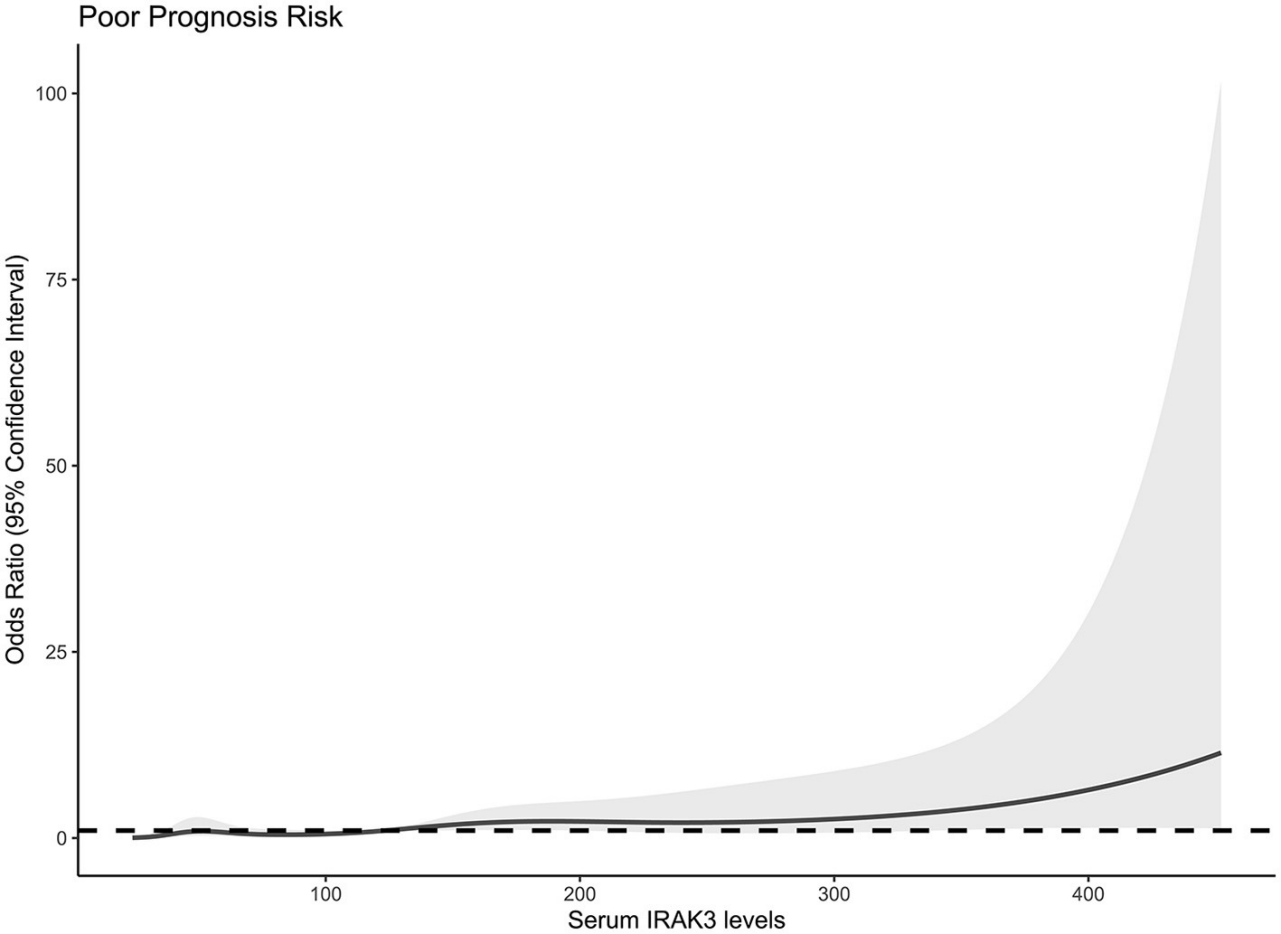
Linear correlation analysis of admission serum interleukin-1 receptor-associated kinase 3 levels with risk of poor prognosis at 6 months after acute ICH. Under restricted cubic spline, admission serum interleukin-1 receptor-associated kinase 3 levels had a linear relation to the risk of poor prognosis 6 months after acute ICH (*P* > 0.05). IRAK3 denotes interleukin-1 receptor-associated kinase 3.

At 6 months after stroke, a total of 75 patients suffered from a poor prognosis (mRS scores of 3–6). As displayed in Figure 5, admission serum IRAK3 levels were substantially higher in patients with poor prognosis than in the remainder (*P* < 0.001), and admission IRAK3 levels highly differentiated between patients with the development of poor prognosis and those with the experience of good prognosis at 6 months following ICH (*P* < 0.001) and levels >99.8 ng/ml distinguished patients at risk of a poor prognosis with the maximum Youden index at 0.422. There was a linear relationship between admission serum IRAK3 levels and the risk of poor prognosis (*P* = 0.168; Figure 6).

In this study, we categorized admission serum IRAK3 levels based on a cutoff value of 99.8 ng/ml. In Table 3, the significant variables differentiating patients with poor prognosis from those with a good prognosis in univariate analyses included age, intraventricular extension of hematoma, NIHSS scores, hematoma volume, early neurological deterioration, blood glucose levels, and admission serum IRAK3 levels > 99.8 ng/ml (all *P* < 0.05).

**Table 3:** Association analysis of 6-month poor prognosis after acute ICH.

**Components**	**Univariate analysis**			**Univariate logistic regression analysis**		
	**Poor prognosis**	**Good prognosis**	***P*** **-value**	**OR**	**95% CI**	***P*** **-value**
Age (years)	62.9 ± 11.0	58.9 ± 11.8	0.032	1.031	1.002–1.061	0.033
Gender (male/female)	40/35	46/31	0.426	0.770	0.405–1.465	0.426
Hypertension	46 (61.3%)	50 (64.9%)	0.645	0.857	0.443–1.657	0.645
Diabetes mellitus	20 (26.6%)	12 (15.6%)	0.094	1.970	0.884–4.387	0.097
Dyslipidemia	27 (36.0%)	18 (23.4%)	0.088	1.844	0.909–3.742	0.090
Cigarette smoking	29 (38.7%)	24 (31.2%)	0.332	1.392	0.713–2.720	0.333
Alcohol drinking	30 (40.0%)	26 (33.8%)	0.426	1.308	0.675–2.532	0.426
Antilipidemic use	21 (28.0%)	14 (18.2%)	0.151	1.750	0.812–3.771	0.153
Anticoagulant use	7 (9.3%)	5 (6.5%)	0.516	1.482	0.449–4.895	0.518
Antiplatelet use	15 (20.0%)	11 (14.3%)	0.350	1.500	0.639–3.520	0.351
Admission time (h)	8.0 (4.7–12.0)	9.0 (5.5–16.5)	0.113	0.958	0.910–1.008	0.097
Blood-collection time (h)	9.0 (6.0–13.0)	11.5 (7.0–18.0)	0.119	0.959	0.912–1.009	0.104
Systolic arterial pressure (mmHg)	139.1 ± 22.8	141.5 ± 21.4	0.497	0.995	0.981–1.010	0.494
Diastolic arterial pressure (mmHg)	83.1 ± 9.6	83.7 ± 8.2	0.663	0.992	0.957–1.028	0.661
Hemorrhagic locations (superficial/deep)	23/52	21/56	0.645	1.179	0.585–2.379	0.645
Intraventricular extension of hematoma	21 (28.0%)	10 (13.0%)	0.022	2.606	1.132–5.999	0.024
Subarachnoidal extension of hematoma	7 (9.3%)	3 (3.4%)	0.176	2.539	0.631–10.215	0.189
NIHSS scores	10 (8–13)	5 (2–10)	<0.001	1.354	1.223–1.498	<0.001
Hematoma volume (ml)	17 (10–24)	7 (5–12)	<0.001	1.126	1.074–1.179	<0.001
Early neurological deterioration	27 (36.0%)	15 (19.5%)	0.023	2.325	1.115–4.849	0.024
Pneumonia	26 (34.7%)	17 (22.1%)	0.085	1.873	0.913–3.841	0.087
Seizure	5 (6.7%)	4 (5.2%)	0.701	1.304	0.336–5.054	0.701
Blood leucocyte count (× 10^9^/l)	7.0 ± 2.2	6.8 ± 2.1	0.599	1.041	0.897–1.208	0.597
Blood glucose levels (mmol/l)	11.0 ± 5.0	9.0 ± 3.4	0.005	1.120	1.033–1.215	0.006
Admission serum IRAK3 levels > 99.8 ng/ml	56 (74.7%)	25 (32.5%)	<0.001	6.131	3.026–12.419	<0.001

Variables were shown as appropriate: count (percentage), mean ± standard deviation, or median (upper-lower quartiles). Data analyses were conducted using the Chi-square test, Fisher exact test, Student's *t*-test, or Mann-Whitney test where appropriate.

NIHSS, National Institutes of Health Stroke Scale; IRAK3, interleukin-1 receptor-associated kinase 3; OR, odds ratio; 95% CI, 95% confidence interval.

Using a binary logistic regression model that incorporated all these significant variables, NIHSS scores (odds ratio, 1.308; 95% confidence interval, 1.121–1.526; *P* = 0.001), hematoma volume (odds ratio, 1.076; 95% confidence interval, 1.019–1.136; *P* = 0.009), and admission serum IRAK3 levels > 99.8 ng/ml (odds ratio, 3.018; 95% confidence interval, 1.261–7.226; *P* = 0.013) independently predicted poor prognosis at 6 months poststroke.

**Figure 7:**
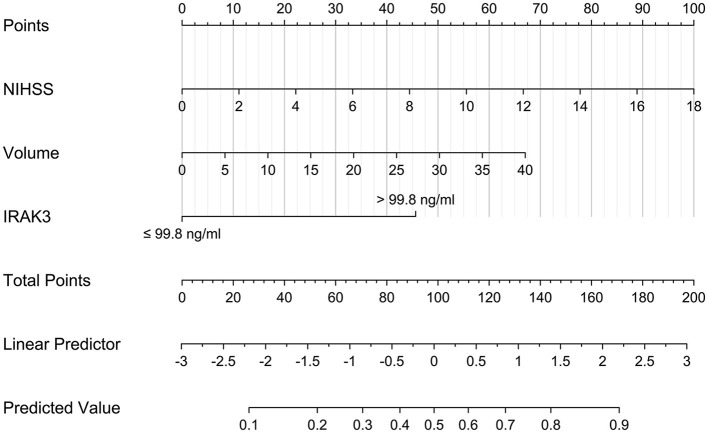
Nomogram describing a 6-month prognosis prediction model in acute ICH. Admission serum IRAK3 denotes interleukin-1 receptor-associated kinase 3, hematoma volume, and National Institutes of Health Stroke Scale scores were combined for discriminating patients at risk of poor prognosis 6 months after acute ICH. IRAK3 denotes interleukin-1 receptor-associated kinase 3.

As shown in Table 4, no significant interactions were found between admission serum IRAK3 levels and other variables, such as age, gender, hypertension, diabetes mellitus, and so on. The prediction model, which included admission serum IRAK3 levels > 99.8 ng/ml, NIHSS scores, and hematoma volume, was illustrated via a nomogram (Figure 7).

**Table 4:** Subgroup analysis for the association of admission serum interleukin-1 receptor-associated kinase 3 with 6-month poor prognosis after acute ICH and interaction analysis between subgroups.

**Subgroup analysis**		**Total (*n*)**	**OR (95% CI)**	***P*-value**	***P*_interaction_-value**
Age	≥65 years	71	2.755 (0.775–9.790)	0.117	0.179
	<65 years	81	4.341 (1.165–16.171)	0.029	
Gender	Male	86	3.303 (1.065–10.243)	0.039	0.228
	Female	66	3.419 (0.893–13.084)	0.073	
Hypertension	Yes	96	2.228 (1.200–4.137)	0.011	0.075
	No	56	3.169 (0.737–12.384)	0.160	
Diabetes mellitus	Yes	32	1.582 (0.037–67.257)	0.810	0.708
	No	120	3.134 (1.226–8.017)	0.017	
Dyslipidemia	Yes	45	2.642 (0.306–22.835)	0.377	0.330
	No	107	3.705 (1.273–10.790)	0.016	
Cigarette smoking	Yes	53	4.726 (0.625–35.727)	0.132	0.260
	No	99	3.264 (1.084–9.831)	0.035	
Alcohol drinking	Yes	56	4.621 (0.576–37.083)	0.150	0.325
	No	96	3.517 (1.111–11.129)	0.032	
Antilipidemic use	Yes	35	1.719 (0.218–13.557)	0.607	0.156
	No	117	3.446 (1.225–9.693)	0.019	
Anticoagulant use	Yes	12	3.857 (0.001–23435.388)	0.761	0.663
	No	140	3.559 (1.408–8.998)	0.007	
Antiplatelet use	Yes	26	7.764 (0.371–162.682)	0.187	0.421
	No	126	2.879 (1.071–7.742)	0.036	
Hemorrhagic locations	Superficial	44	7.537 (1.026–55.375)	0.047	0.170
	Deep	108	2.560 (0.869–7.540)	0.088	
Intraventricular extension of hematoma	Yes	31	2.040 (0.277–15.038)	0.484	0.324
	No	121	3.464 (1.290–9.300)	0.014	
Subarachnoidal extension of hematoma	Yes	10	3.861 (0.068–219.656)	0.512	0.241
	No	142	3.071 (1.257–7.501)	0.014	
Early neurological deterioration	Yes	42	4.366 (0.805–23.669)	0.088	0.059
	No	110	2.707 (1.121–7.624)	0.012	
Pneumonia	Yes	43	1.209 (0.211–6.916)	0.831	0.624
	No	109	3.918 (1.381–11.120)	0.010	
Seizure	Yes	9	8.379 (0.112–624.865)	0.334	0.144
	No	143	2.985 (1.309–6.804)	0.009	

Associations were adjusted for age, intraventricular extension of hematoma, National Institutes of Health Stroke Scale scores, hematoma volume, early neurological deterioration, and blood glucose levels.

OR, odds ratio; 95% CI, 95% confidence interval.

**Figure 8:**
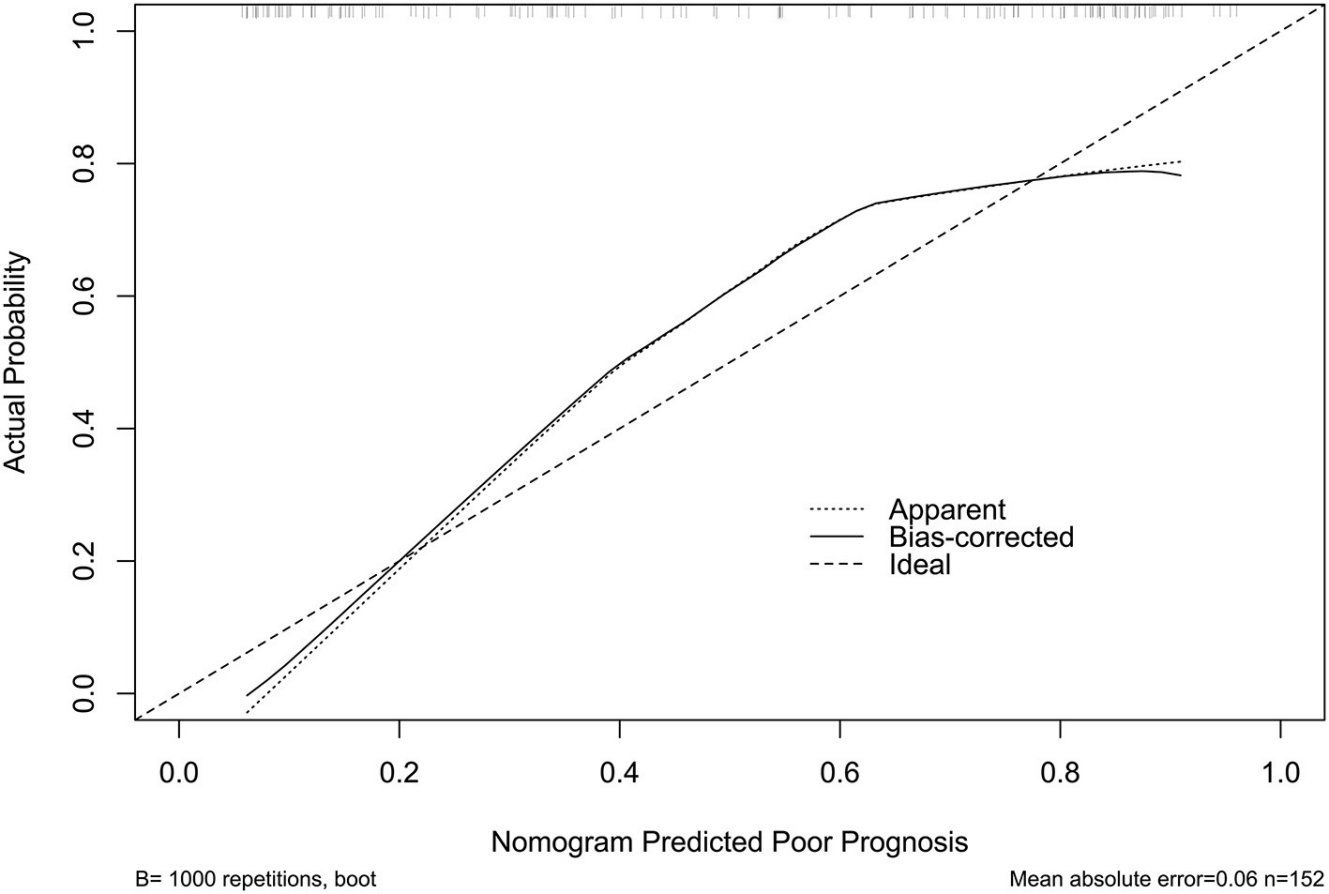
Stability assessment of the nomogram of poor prognosis at poststroke 6 months. Using calibration curve analysis, the nomogram took possession of stability for predicting a 6-month poor prognosis after acute ICH.

**Figure 9:**
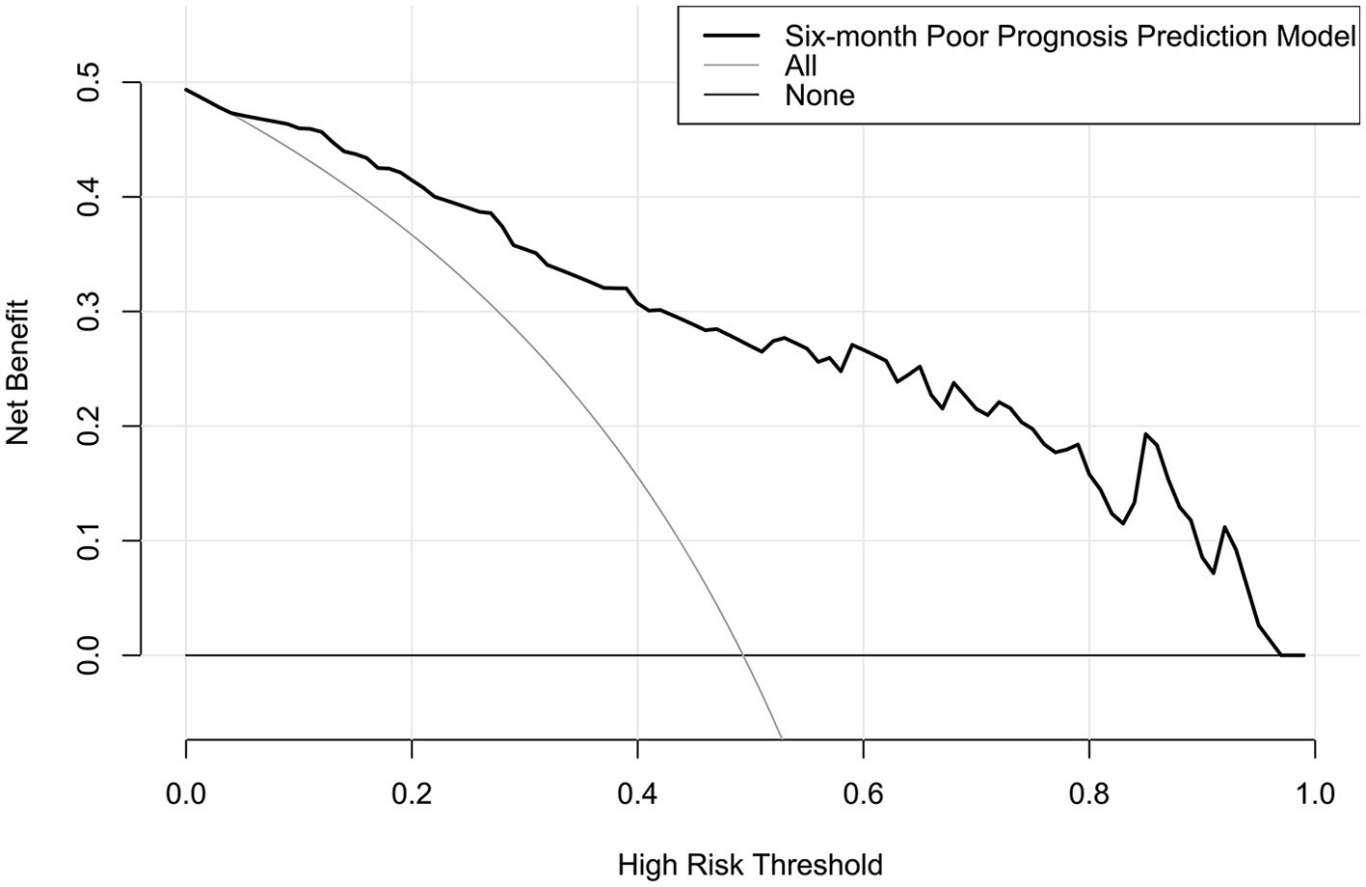
Decision curve for clinical benefit assessment for nomogram of poor prognosis at 6 months after acute ICH. The nomogram was at the state of good clinical capability for predicting poor prognosis at 6 months after acute ICH.

**Figure 10:**
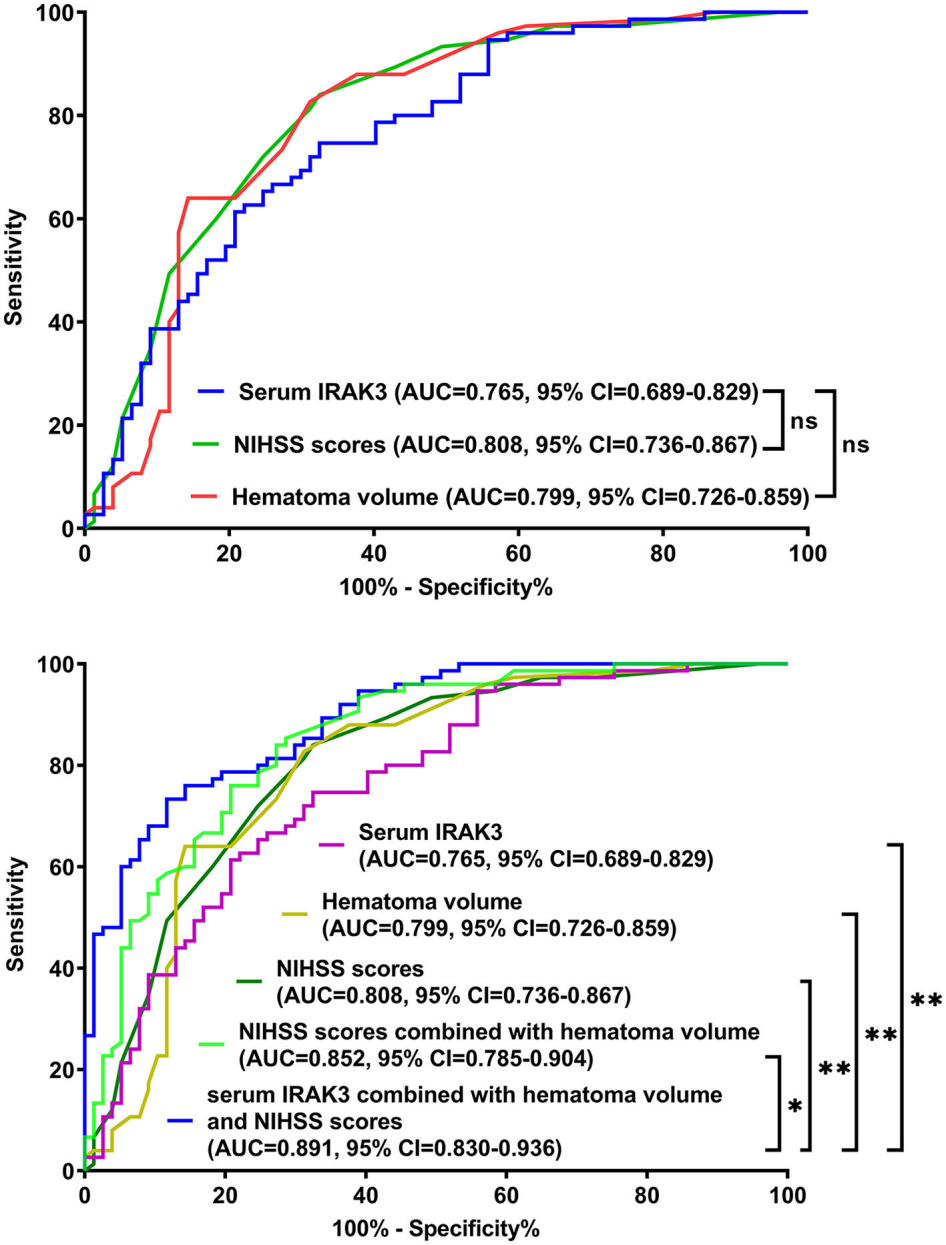
Receiver operating characteristic curves for assessing prognostic predictive ability in patients with acute ICH. Regarding area under the curve, the prognostic predictive ability was similar between admission serum interleukin-1 receptor-associated kinase 3 levels, National Institutes of Health Stroke Scale scores, and hematoma volume (all *P* > 0.05). The combination model, in which admission serum interleukin-1 receptor-associated kinase 3, National Institutes of Health Stroke Scale scores, and hematoma volume were integrated, had significantly the highest prognostic predictive ability compared to other indicators (all *P* < 0.05). IRAK3, interleukin-1 receptor-associated kinase 3; NIHSS, National Institutes of Health Stroke Scale; AUC, area under the curve; 95% CI, 95% confidence interval; ns, non-significant. *P < 0.05; **P < 0.01.

The model performed well in calibration curve analysis (Figure 8) and decision curve analysis (Figure 9). In Figure 10, there was no statistically significant difference in AUC between admission serum IRAK3 levels and NIHSS scores or hematoma volume (both *P* > 0.05). However, the model exhibited the highest AUC among all indicators, including admission serum IRAK3 levels, NIHSS scores, hematoma volume, and the combination of NIHSS scores and hematoma volume (all *P* < 0.05).

## 4 Discussion

A recent clinical study showed that, compared to healthy controls, serum IRAK3 levels were substantially elevated at admission in patients with severe traumatic brain injury (14). In our study, we investigated longitudinal change in serum IRAK3 levels in a portion of patients with ICH. Our finding was that serum IRAK3 levels were immediately increased after ICH, with the highest levels on days 1 and 3, and afterward were gradually decreased until day 15, and the levels during 15 days were substantially higher than those of controls. In the central nervous system, IRAK3 could be mainly secreted from microglia, and its expressions were prominently upregulated after brain injury (11–13). Thus, it is assumed that serum IRAK3 may be at least partially derived from the central nervous system. Since IRAK3 could be produced from peripheral blood cells, such as neutrophils and macrophages (17–19), at least a portion of serum IRAK3 may be derived from the peripheral system. To verify such a postulation, mRNA determinations should be made in the future.

IRAK3 is known to exert inhibitory effects on inflammatory responses (20–22). Moreover, compelling evidence has shown that IRAK3 may also confer neuroprotective effects (11–13). Specifically, IRAK3 has been shown to substantially suppress neuroinflammation in mice with autoimmune encephalomyelitis, middle cerebral artery occlusion, or sub-acute Parkinson's disease, thereby reducing brain edema, improving the blood–brain barrier, decreasing neuronal death, and finally recovering neurological function (11–13).

In other words, IRAK3 could be considered a promising new therapeutic target for central nervous system diseases. Moreover, the production of IRAK3 may be part of a compensatory response to acute brain injury (14), indicating that the significant elevation of serum IRAK3 levels after ICH may be linked to this protective mechanism. However, while ICH is an acute brain injury disease with mechanisms of secondary brain injury potentially related to anti-inflammatory processes, the specific mechanisms underlying IRAK3's role require further investigation.

In the current study, increased admission serum IRAK3 levels had an independent correlation with NIHSS scores and hematoma volume. Similarly, in a recent study of severe traumatic brain injury, rising serum IRAK3 levels were independently related to trauma severity, which was reflected by Glasgow coma scale scores and Rotterdam classification scores (14). Alternatively, serum IRAK3 could independently predict a poor 6-month prognosis after ICH in our study. In patients with severe traumatic brain injury, serum IRAK3 also independently distinguished poor prognosis at 6 months after head trauma (14). Thus, serum IRAK3 levels may be highly correlated with illness severity and clinical outcomes after acute brain injury. In our study, we found that admission serum IRAK3 levels were linearly correlated with the risk of poor prognosis under restricted cubic spline, supporting that it is rational that admission serum IRAK3 was transformed into a categorical variable. We further performed subgroup analysis and revealed no interactions existed between admission serum IRAK3 levels and other conventional factors, such as age, gender, hypertension, diabetes mellitus, and so on. Taken together, such data strongly support the presumption that serum IRAK3 may be a potential prognostic biomarker of ICH.

NIHSS and hematoma volume have been believably accepted as the two indicators of prognosis prediction in ICH patients (23–25). Our study showed that NIHSS and hematoma volume were independently associated with the poor prognosis of ICH patients. Moreover, under the ROC curve, the prognosis prediction ability was similar between admission serum IRAK3 levels, NIHSS scores, and hematoma volume. The combined logistic regression method merged NIHSS scores, hematoma volume, and admission serum IRAK3 levels > 99.8 ng/ml (cutoff value) into a prediction model. The model had significantly the highest AUC compared to NIHSS scores, hematoma volume, admission serum IRAK3, and a combination of NIHSS scores with hematoma volume. Moreover, the model performed well using a series of statistical methods, such as the calibration curve and decision curve. These results also support the notion that serum IRAK3 may serve as a potential biomarker for predicting the neurological outcome of ICH. Conversely, the combinational use of serum IRAK3 measurements and assessments of NIHSS and hematoma volume may be a better choice for assistance with clinical work in ICH management.

There are several strengths and weaknesses. The strengths are that (1) this may be the first series to investigate admission serum IRAK3 as a prognostic predictor of ICH and subsequently demonstrate its independent relation to severity and prognosis of ICH; (2) the ICH severity correlation and prognosis association with admission serum IRAK3 levels were all verified using multivariate analyses in the current study, and hence the conclusions may be more reliable and scientific. The weaknesses are that (1) although we uncovered dynamic change in serum IRAK3 levels after ICH, their AUCs were not compared at multiple time points because of the small sample size; (2) the conclusions with respect to the prognostic importance of serum IRAK3 in ICH may be made, but a further validation should be necessary in a larger cohort study; (3) the current study assessed serum IRAK3 levels up to 15 days after ICH and linked these levels to 6-month outcomes. However, additional longitudinal follow-ups beyond 6 months would be valuable to understand the long-term trajectory of IRAK3 levels and their predictive value over time. Assessing whether IRAK3 levels would normalize or remain elevated in the long term and how this would be correlated with chronic outcomes could provide important insights for disease management and its prognosis. (4) In this study, healthy volunteers were consecutively chosen to constitute a control group. They were free from hypertension, diabetes mellitus, chronic renal disease, and so on. Moreover, they had normal results in blood white blood cell counts, blood sugar levels, blood platelet counts, red blood cell counts, and so on. Although age, gender, cigarette smoking, and alcohol drinking were matched between patients and controls, other variables, such as hypertension, diabetes mellitus, and chronic renal disease, may be confounding factors that may affect serum IRAK3 levels. Therefore, it is better that a large number of controls could be enrolled and inclusion of numerous potential confounding factors could be implemented in the future; (5) systemic infection, including sepsis, could influence circulation levels of IRAK3 (22), and nevertheless, this potential confounding parameter was uninvestigated in these patients with ICH, hinting that a further study with incorporation of systemic infection as a recordable variable may be preferred in future studies.

## 5 Conclusion

Serum IRAK3 levels are increased early after ICH and may remain elevated until day 15. Notably, admission serum IRAK3 levels show a strong correlation with NIHSS scores and hematoma volume and are independently associated with 6-month mRS scores and poor prognosis after ICH. A predictive model incorporating NIHSS scores, hematoma volume, and admission serum IRAK3 levels demonstrates high discriminatory efficiency in assessing the risk of poor outcomes. In summary, serum IRAK3 appears to be a promising biochemical marker for evaluating the severity and predicting the prognosis of ICH.

## Data Availability

The raw data supporting the conclusions of this article will be made available by the authors, without undue reservation.
